# Diet was less significant than physical activity in the prognosis of people with sarcopenia and metabolic dysfunction-associated fatty liver diseases: Analysis of the National Health and Nutrition Examination Survey III

**DOI:** 10.3389/fendo.2023.1101892

**Published:** 2023-02-23

**Authors:** Yun Yi, Chun Wang, Yang Ding, JiangHua He, YuQing Lv, Ying Chang

**Affiliations:** ^1^ Department of Gastroenterology, Zhongnan Hospital of Wuhan University, Wuhan, China; ^2^ Hubei Clinical Center and Key Laboratory of Intestinal and Colorectal Diseases, Zhongnan Hospital of Wuhan University, Wuhan, China

**Keywords:** sarcopenia, MAFLD, mortality, physical activity, nutrition, NHANES

## Abstract

**Background:**

Sarcopenia is prevalent in metabolic dysfunction-associated fatty liver diseases (MAFLD), and the primary treatment for both diseases is lifestyle modification. We studied how dietary components and physical activity affect individuals with sarcopenia and MAFLD.

**Materials and methods:**

We conducted a study utilizing National Health and Nutrition Examination Survey (NHANES) III (1988–1994) data with Linked Mortality file (through 2019). The diagnosis of fatty liver disease (FLD) was based on ultrasound images revealing moderate and severe steatosis. Using bioelectrical measures, sarcopenia was assessed. Using self-report data, dietary intake and physical activity levels were evaluated.

**Results:**

Among 12,259 participants, 2,473 presented with MAFLD, and 290 of whom had sarcopenia. Higher levels of physical activity (odds ratio [OR] = 0.51 [0.36–0.95]) and calorie (OR = 0.58 [0.41–0.83]) intake reduced the likelihood of sarcopenia in MAFLD patients. During a median follow-up period of 15.3 years, 1,164 MAFLD and 181 MAFLD patients with sarcopenia perished. Increased activity levels improved the prognosis of patients with sarcopenia (Insufficiently active, HR = 0.75 [0.58–0.97]; Active, HR = 0.64 [0.48–0.86]), which was particularly pronounced in older patients.

**Conclusion:**

In the general population, hyperglycemia was highly related to MAFLD prognosis. Physical inactivity and a protein-restricted diet corresponded to sarcopenia, with physical inactivity being connected to poor outcomes. Adding protein supplements would be beneficial for older people with sarcopenia who are unable to exercise due to frailty, while the survival benefits were negligible.

## 1 Introduction

Non-alcoholic fatty liver disease (NAFLD), initially defined as fatty liver disease in the absence of significant alcohol intake and other causes of steatosis, is a common liver disorder that is strongly associated with features of the metabolic syndrome (1). With a prevalence of approximately 25% in the general population, NAFLD has emerged as a leading cause of advanced liver disorders, posing an underestimated global healthcare burden (2). However, the term “non-alcoholic” overemphasized the absence of alcohol consumption while underemphasizing the significance of metabolic factors, which are the primary drivers of the course of the disease (3). It has been suggested that metabolic (dysfunction)-associated fatty liver disease (MAFLD), which endorsed a list of positive diagnostic criteria and offered a more comprehensive description of its metabolic-related natural courses, may represent the importance of metabolic risk factors and improve the detection of the disease (4, 5). Despite the rising prevalence and increasing impact of MAFLD (5, 6), there is an absence of approved pharmacotherapy for this significant condition, whose treatment remains limited to lifestyle modification (7, 8).

Sarcopenia is a geriatric syndrome characterized by generalized loss of muscle mass and its function, and is associated with adverse outcomes (9, 10). Since age-related sarcopenia is inevitable, inactivity and poor diet can accelerate the process. Physical inactivity may contribute to the development of sarcopenia (11, 12), and an increase in moderate-to-vigorous physical activity levels could potentially prevent sarcopenia from developing (13). A cohort study demonstrated that malnutrition is related to a fourfold increased risk of developing sarcopenia over a four-year follow-up period (14).Moreover, lean muscle mass in older individuals is positively associated with protein consumption (15), where insufficient protein intake and a lack of amino acid availability contribute to deficits in muscle protein synthesis (16). Physical exercise has a protective effect on muscle mass and function maintenance, in comparison, the effect of supplemental nutrition on muscle function is uncertain (17–19). A number of studies have revealed that dietary supplements may enhance the benefit of exercise training despite the relatively low quality of the evidence (20); however, the existing evidence for nutrition interventions is based on groups with varying ages, frailties, and nutritional conditions, and the findings are inconsistent (21, 22). Currently, large scale clinical trials are addressing the role of exercise and nutritional interventions in the treatment of sarcopenia, such as the European SPRINTT trial (NCT02582138) (23). In addition to aforementioned variables, sarcopenia is secondary with chronic illness, such as liver diseases, renal diseases, inflammatory diseases, and malignancies (24). Recent studies have observed a significantly higher prevalence of sarcopenia among obese and NAFLD patients (25–27). Multiple potential mechanisms evolved in the link between sarcopenia and NAFLD, including insulin resistance, elevated inflammation, myokines secreted by skeletal muscle, vitamin D deficiency and physical inactivity, but the specific mechanism is yet unclear (28).

Lifestyle modification remains the first-line intervention for fatty liver diseases (FLD), and a standard approach consists of a 7%–10% weight loss from baseline. Similarly, there are no approved pharmacological treatment for sarcopenia. In liver cirrhosis, the severity of sarcopenia increased as the liver disease progress (29), which was primarily regarded as a sign of malnutrition and required nutritional supplementation. However, these treatments had minimal benefits for survival improvement (30).

The Third National Health and Nutrition Examination Survey (NHANES III) was a well-designed population-based program, collecting data from US adults from 1988 to 1994. In this context, we aim to analyze the associations between diet, physical activity and sarcopenic MAFLD using the population-based survey data.

## 2 Material and methods

### 2.1 Data source and population

National Health and Nutrition Examination Survey (NHANES) is a population-based survey program carried out by the National Center for Health Statistics (NCHS), which aims to evaluate the health and nutritional status of civilian, non-institutionalized members in the US population (31). Our work is predicated on the database of NHANES III (1988–1994) (32), which is the only survey that recorded liver ultrasonography data using a Toshiba Sonolayer SSA-90A and Toshiba video recorders (33). The steatosis severity of participants was reevaluated and graded by experts between 2009 and 2010, and FLD was defined as moderate or severe hepatic steatosis based on hepatic ultrasound imaging. Household interviews were conducted by qualified health technicians utilizing a computer-assisted personal interview system to collect data on demographic variables and health history. Body mass index (BMI) was computed by dividing weight in kilograms by height in meters squared, rounding to the nearest decimal. The Linked Mortality Files (LMF) have been updated with mortality follow-up data through December 31, 2019 (34). During the follow-up phase, respondents without matched death records were presumed alive. Survival time was counted from a subject who participated in the survey to death or December 31, 2019. Informed consent was obtained from all participants, and ethical approval was obtained from the NCHS Ethics Review Board.

**Figure 1:**
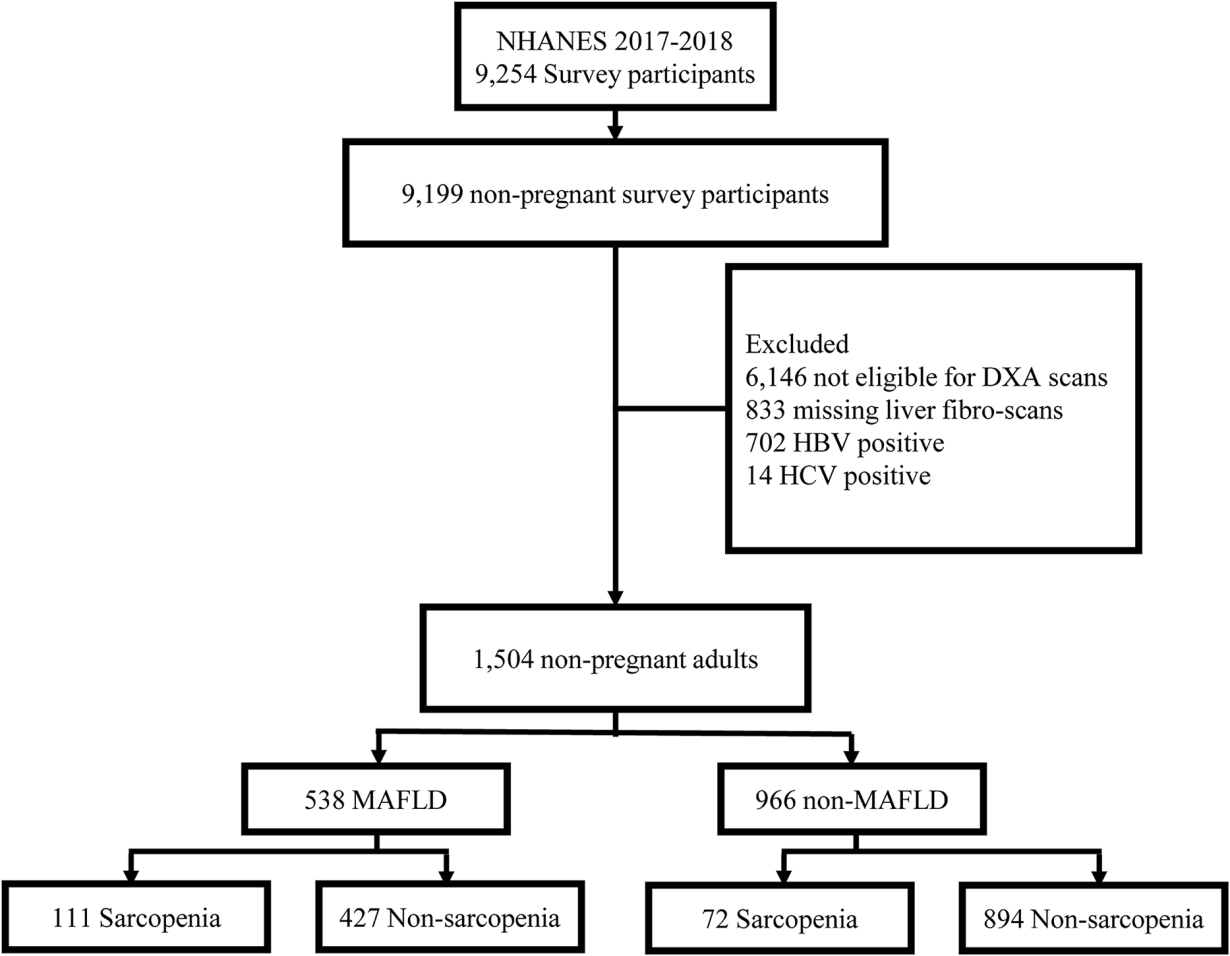
Flow Chart of Participants for the Study.

A total of 20,050 subjects were included in the NHANES III survey. Among these subjects, 7,791 were excluded based on the following criteria (1): missing data of BIA (n=4186); (2) missing data of height or weight (n=25); (3) positive serologic markers for hepatitis B (n=73) or C (n=348) virus; (4) patients with missing data of liver ultrasounds (n=3159); After applying the above exclusion criteria, we included 12,259 subjects aged 18 to 75 years, of which 2,473 were MAFLD patients, and 9,786 were non-MAFLD patients (**Figure 1**).

### 2.2 Definition of MAFLD

MAFLD was diagnosed in individuals with FLD and any of the following three medical conditions: overweight/obesity (body mass index [BMI] ≥ 25 kg/m^2^), type 2 diabetes mellitus (T2DM), or the existence of metabolic dysregulation (5). Metabolic dysregulation was defined by the presence of at least two metabolic risk abnormalities: (a) waist circumference ≥ 102 cm in men and ≥ 88 cm in women; (b) blood pressure ≥ 130/85 mmHg or specific drug treatment; (c) TG ≥ 150 mg/dL or specific drug treatment; (d) HDL-C < 40 mg/dL for men and < 50 mg/dL for women; (e) prediabetes (FPG = 100–125 mg/dL or HbA1c = 5.7%–6.4%); (f) homeostasis model assessment of insulin resistance score (HOMA-IR) ≥ 2.5; and/or (g) CRP > 2 mg/L. The classification of individuals into MAFLD and non-MAFLD categories was based on their diagnoses.

### 2.3 Definition of sarcopenia

Following the recommendation of 2^nd^ edition of European Working Group on Sarcopenia in Older People (EWGSOP2), this study employs bioelectrical impedance analysis (BIA) to diagnose sarcopenia based on the existence of decreased muscle quantity or quality (9). For the NHANES III database, BIA was measured as the resistance at 50 kHz between the right wrist and ankle of a supine participant using A Valhalla 1990B Bio-Resistance Body Composition Analyzer (Valhalla Medical, San Diego, California, USA).

Here, Skeletal muscle mass (SMM) was calculated by BIA from NHANES III database using Janssen’s equation: SMM (kg)= (height in cm)^2^/BIA-resistance × 0.401 + (sex × 3.825) + (age in years × −0.071) + 5.102, where BIA-resistance is measured in ohms, and sex is encoded as 1 for male and 0 for female (35). Using the following formula, skeletal muscle mass index (SMI) was calculated: SMI = skeletal muscle mass in kg/body weight in kg × 100. Participants were considered to have sarcopenia if their SMI was more than two standard deviation below the sex-specific mean for young adults aged 18 to 39 (9, 35).

### 2.4 Physical activity level

Physical activity questionnaires were given at a home interview for all participants, inquiring about the frequency of leisure time activities (walking, running or jogging, riding, swimming, aerobics, dancing, etc.) in the previous month. The intensity of each activity was evaluated by metabolic equivalent (MET) based on the criteria from the Compendium of Physical Activities (36), which defines one MET as the energy expended at resting metabolic rate.

The NHANES III datasets collected information on the intensity rating and frequency of each individual’s daily physical activity. The activities are classified into moderate (METs ranging from 3 to 6) and vigorous (METs above 6) categories based on their intensity rates. Active group was characterized as those who engaged in moderate or vigorous activity at least five or three times per week. The inactive group was defined as those who participate in no physical activity during their leisure time. The insufficiently active group fell in the middle between active and inactive levels of physical activity (37, 38).

### 2.5 Ascertainment of nutrient components intake

A nutritional interview comprising a 24-hour recall of dietary intake was conducted, with participants providing information on specific foods and quantities. Following the instruction of the Nutrient Composition Data Bank, the grams of nutrient components (carbohydrate, protein, fat, cholesterol, saturated fatty acids, monounsaturated fatty acid, and polyunsaturated fatty acid) were recorded and calculated.

In our study, the absolute quantity and percentage of energy intake from each macronutrient were categorized into gender-specific quartiles (Q1, Q2, Q3, and Q4). Additionally, the contribution of carbohydrates, proteins, and fatty acids to the overall amount of energy intake (% of total energy consumed) was calculated. The quartile variables were modeled as dummy variables, comparing each quartile to the lowest one (Q1).

In accordance with the American Gastroenterological Association’s (AGA) guidelines for lifestyle modification for NAFLD management, we further grouped individuals based on their calorie and protein intake (7). The definition of a hypocaloric diet was < 1200 kcal/day for women and < 1500 kcal/day for men. In addition, the relative daily protein intake of participants was graded as low (< 1.2 g/kg), adequate (1.2–1.5 g/kg), and high (> 1.5 g/kg) based upon recommendations for patients with sarcopenia.

### 2.6 Other definitions

Household interviews were conducted by skilled interviewers utilizing a computer-assisted personal interview system to collect data on demographic variables and health history. The data on body measurements were gathered by qualified health technicians. Body mass index (BMI) was computed by dividing weight in kilograms by height in meters squared, and then rounding to the nearest decimal. Participants were asked to fast for 9 hours before the blood sample was collected. Serum insulin and plasma glucose concentrations were measured by radioimmunoassay and a hexokinase enzymatic array from fasting blood samples. The HOMA-IR score was determined by the following formula: HOMA-IR = (Fasting insulin in μIU/mL) × (Fasting glucose in mg/dL)/405 (39). In addition, concentrations of alanine aminotransferase (ALT), aspartate aminotransferase (AST), creatinine, gamma-glutamyl transferase (GGT), total bilirubin, albumin, glycated hemoglobin (HbA1c), low-density lipoprotein cholesterol (LDL-C), triglyceride (TG), high-density lipoprotein cholesterol (HDL-C), and C-reactive protein (CRP) were measured. Details of measurements are available at http://www.cdc.gov/nchs/nhanes/index.htm.

T2DM was defined by a self-reported diabetic medical history, an FPG ≥ 126 mg/dL, or an HbA1c of ≥ 6.5%. Hypertension (HTN) was defined by self-reported medical history of HTN, systolic blood pressure readings above 130 mmHg, or diastolic blood pressure measures above 80 mmHg from an average of 3 measurements. Hyperlipidemia (HL) was defined by a reported history of HL, cholesterol ≥ 200 mg/dL, LDL-C ≥ 130 mg/dL, or HDL-C ≤ 40 mg/dL for men and ≤ 50 mg/dL for women.

NAFLD Fibrosis Score (NFS) score is a non-invasive method to separate NAFLD patients with and without advanced fibrosis, calculated as: NFS = -1.675 + (0.037 × Age in years) + (0.094 × BMI in kg/m2) + (1.13 × Impaired fasting glucose or diabetes) + (0.99 × AST in U/L/ALT in U/L) – (0.013 × Platelets in ×10^9^/L) – (0.66 × Albumin in g/dL), where impaired fasting glucose/diabetes is encoded as 1 and 0 for participants with or without abnormal fasting glucose (40). Fibrosis-4 (FIB-4) index was designed to predict significant fibrosis in a simple equation: (Age in years × AST in U/L)/(Platelets in ×10^9^/L × ALT^0.5^ in U/L) (41). Advanced fibrosis was determined by a NFS > 0.675 (40) or Fibrosis-4 (FIB-4) index > 2.67 (41).

### 2.7 Statistical analysis

We compared the baseline characteristics of MAFLD and non-MAFLD participants using data from NHANES III. Continuous variables were expressed as means ± standard deviation (SD), while categorical variables were expressed as percentages. The Student t-test was utilized for normally distributed variables, the Chi-squared test for categorical variables, and the Mann-Whitney U-test for non-normally distributed variables. Multivariate logistic regression models adjusted for confounders were used to evaluate the association between sarcopenia and other clinical covariates. In tests of interaction, age (dichotomized into < 60 years and ≥ 60 years) modified the effect of sarcopenia, whereas gender did not interact significantly with sarcopenia. Cox proportional hazards models were developed to estimate hazard ratios (HR) and 95% confidence intervals (CI) of risk factors for all-cause mortality in participants with sarcopenia or MAFLD. Model 1 was adjusted for age, sex, race, and BMI levels. Model 2 was adjusted for age, sex, race, BMI levels, and the existence of advanced fibrosis. Model 3 was adjusted for variables mentioned in model 2 with T2DM. Model 4 was adjusted for all variables in model 3 with other medical histories (HTN, HL, smoking). No evident interactions between MAFLD and sarcopenia were found (p > 0.05). All tests were two-tailed, and a p value less than 0.05 was considered statistically significant. R 4.2.0 (https://www.r-project.org/) was used to conduct all analyses.

## 3 Results

### 3.1 Data characteristics

**Figure 2:**
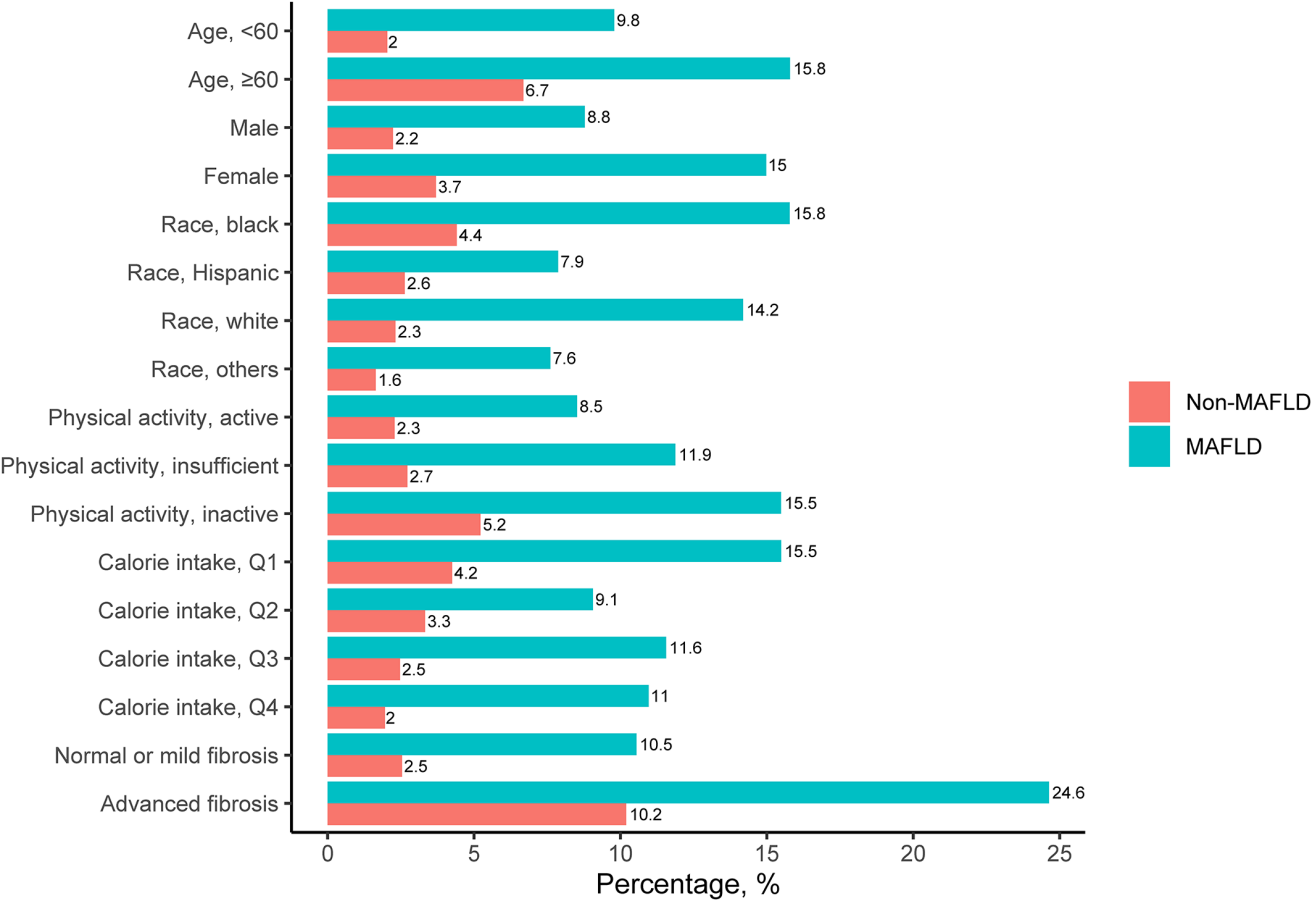
Prevalence of Sarcopenia among Participants with and without MAFLD. *MAFLD, metabolic-associated fatty liver diseases.*.

A total of 12,259 participants from NHANES III data sets were included in this analysis, of whom 2,473 (20.2%) were diagnosed with MAFLD (**Figure 1**). The included participants contained 5,862 (47.8%) males aged 43.8 ± 15.9 years. Individuals with MAFLD had a higher prevalence of sarcopenia than those without MAFLD (11.7% vs. 3.0%), and this tendency persisted regardless of age, sex, ethnicity, levels of physical activity, calorie consumption, and liver fibrosis (**Figure 2**). The statistical differences between MAFLD and non-MAFLD groups were listed in **Table S3**.

The demographic, laboratory, and lifestyle characteristics of participants were demonstrated in **Tables S1–2**, categorized by the presence of MAFLD and sarcopenia. Sarcopenia, with or without MAFLD, was characterized by female gender, advanced age, and central obesity. Moreover, self-report data demonstrated that those with sarcopenia consumed fewer calories and engaged in less physical activity than those without the condition.

### 3.2 Identify risk factors for sarcopenia among MAFLD participants

The fully-adjusted logistic regression model showed that the presence of MAFLD was associated with an increased risk of sarcopenia (odds ratio [OR] = 1.38 [95% CI 1.11–1.73]) (**Table 1**). We then generated multivariate Logistic regression models (adjusted for age, sex, and race) to identify sarcopenia-related factors by calculating their ORs amongst the MAFLD population. As shown in **Table 2**, sarcopenia was associated with physical activity levels (active vs. inactive, OR=0.51 [95% CI 0.36–0.95]), calorie intake (Q2 vs. Q1, OR = 0.58 [95% CI 0.41–0.83]), carbohydrates (Q2 vs. Q1, OR = 0.54 [95% CI 0.37–0.76]), and fatty acids (Q2 vs. Q1, OR = 0.62 [95% CI 0.44–0.89]) intake.

**Table 1:** Multivariate Analysis for Sarcopenia in overall population.

	OR (95% CI)	P-value
MAFLD	1.38 (1.11–1.73)	0.004
Age	1.06 (1.05–1.07)	< 0.001
Male sex	1.37 (1.09–1.73)	0.006
Race		
Black	Reference	
Hispanic	0.81 (0.61–1.07)	0.144
White	0.83 (0.64–1.09)	0.182
Others	0.62 (0.31–1.25)	0.181
BMI	1.34 (1.31–1.36)	< 0.001
HbA1c	0.92 (0.85–0.99)	0.088
Physical activity		
Inactive	Reference	
Insufficiently active	0.79 (0.61–1.03)	0.085
Active	0.71 (0.53–0.95)	0.02
Calorie ^a^		
Q1	Reference	
Q2	0.84 (0.64–1.11)	0.226
Q3	0.82 (0.61–1.10)	0.177
Q4	0.61 (0.44–0.85)	0.003

a Q1: 0–1763 kcal in male, 0–1230 kcal in female; Q2: 1764–2365 kcal in male, 1231–1647 kcal in female; Q3: 2366–3128 kcal in male, 1648–2148 kcal in female; Q4: >3128 kcal in male, >2148 kcal in female.

MAFLD, metabolic dysfunction-associated fatty liver diseases; BMI, body mass index; HbA1c, glycosylated hemoglobin.

**Table 2:** Age, Sex and Race-adjusted Odds Ratio (OR) for Sarcopenia in patients with and without MAFLD.

Covariates	MAFLD		Non-MAFLD	
	OR (95% CI)	P-value	OR (95% CI)	P-value
Physical activity				
Inactive	Reference		Reference	
Insufficient	0.77 (0.57–1.04)	0.089	0.65 (0.49–0.87)	0.004
Active	0.51 (0.36–0.95)	< 0.001	0.57 (0.42–0.77)	< 0.001
Calorie ^a^				
Q1	Reference		Reference	
Q2	0.58 (0.41–0.83)	0.003	1.00 (0.73–1.36)	0.978
Q3	0.82 (0.58–1.14)	0.238	0.86 (0.61–1.21)	0.388
Q4	0.83 (0.58–1.20)	0.333	0.83 (0.57–1.20)	0.313
Protein ^b^				
Q1	Reference		Reference	
Q2	0.98 (0.70–1.38)	0.913	0.98 (0.71–1.34)	0.898
Q3	0.83 (0.57–1.20)	0.325	0.85 (0.61–1.19)	0.335
Q4	1.04 (0.73–1.48)	0.838	0.94 (0.66–1.34)	0.729
Carbohydrates ^c^				
Q1	Reference		Reference	
Q2	0.53 (0.37–0.76)	< 0.001	0.85 (0.61–1.17)	0.309
Q3	0.75 (0.53–1.05)	0.09	1.12 (0.82–1.53)	0.471
Q4	0.76 (0.53–1.08)	0.128	0.64 (0.43–0.94)	0.024
Fatty acids ^d^				
Q1	Reference		Reference	
Q2	0.62 (0.44–0.89)	0.009	1.01 (0.74–1.38)	0.949
Q3	0.89 (0.64–1.25)	0.503	0.86 (0.61–1.21)	0.387
Q4	0.82 (0.57–1.19)	0.297	1.02 (0.72–1.44)	0.928
Carbohydrates %				
<40%	Reference		Reference	
40%-59%	0.82 (0.60–1.13)	0.223	0.89 (0.65–1.22)	0.484
≥60%	0.85 (0.57–1.28)	0.434	1.04 (0.71–1.52)	0.832
Fatty acids %				
<40%	Reference		Reference	
≥40%	1.18 (0.89–1.57)	0.257	1.09 (0.83–1.43)	0.549
Relative protein intake				< 0.001
< 1.2 g/kg	Reference		Reference	
1.2–1.5 g/kg	0.28 (0.14–0.53)	< 0.001	0.06 (0.03–0.14)	< 0.001
>1.5 g/kg	0.39 (0.23–0.68)	< 0.001	0.27 (0.17–0.43)	< 0.001

a Q1: 0–1763 kcal in male, 0–1230 kcal in female; Q2: 1764–2365 kcal in male, 1231–1647 kcal in female; Q3: 2366–3128 kcal in male, 1648–2148 kcal in female; Q4: > 3128 kcal in male, > 2148 kcal in female.

b Q1: 0-64.0 g in male, 0-44.0 g in female; Q2: 64.1-89.2 g in male, 44.1-62.1 g in female; Q3: 89.3–121.0 g in male, 62.2-82.9 g in female; Q4: > 121.0 g in male, > 83.0 g in female.

c Q1: 0–203 g in male, 0–152 g in female; Q2: 204–280 g in male, 153–206 g in female; Q3: 281–371 g in male, 207–271 g in female; Q4: > 371 g in male, > 271 g in female.

d Q1: 0-58 g in male, 0-40 g in female; Q2: 59-87 g in male, 41-60 g in female; Q3: 88–124 g in male, 61-87 g in female; Q4: > 124 g in male, > 87 g in female.

Adjusted for age, sex, and race.

MAFLD, metabolic dysfunction-associated fatty liver diseases.

Ordinal logistic regressions were performed to further reveal the relationship between sarcopenia and lifestyle factors. Sarcopenia was significantly and negatively associated with higher levels of physical activity (OR = 0.74 [95% CI 0.62–0.87]) (**Table S4**) and appropriate relative protein intake (OR = 0.48 [95% CI 0.35–0.65]) (**Table S5**). In contrast, there was no connection between sarcopenia and absolute calorie, carbohydrates, protein, or fat consumption (**Tables S6–7**).

### 3.3 All-cause mortality

Of the overall NHANES III cohort (1988–1994), 290 (2.37%) patients presented with MAFLD and sarcopenia, of whom 181 (62.41%) individuals died after a median follow-up of 15.3 years.

Analyses of the relationships between MAFLD and sarcopenia and all-cause mortality were conducted using Models 1 through 4, which included age, sex, race, health behavior, and medical history as adjustments (**Table 3**). The presence of sarcopenia was associated with a poorer prognosis after modifications, whereas the presence of MAFLD was unable to predict survival when a history of T2DM was added to the model (Models 3–4).

**Table 3:** Hazard Ratios of Risk Factors for All-cause Mortality (Multiple Imputation Analysis).

Covariate	Model 1		Model 2		Model 3		Model 4	
	HR (95% CI)	P-value	HR (95% CI)	P-value	HR (95% CI)	P-value	HR (95% CI)	P-value
MAFLD	1.11 (1.03–1.19)	0.007	1.11 (1.04–1.20)	0.003	1.05 (0.97–1.13)	0.209	1.03 (0.96–1.11)	0.427
Sarcopenia	1.18 (1.06–1.34)	0.003	1.17 (1.04–1.30)	0.009	1.16 (1.03–1.30)	0.013	1.14 (1.02–1.28)	0.025
Age	1.09 (1.09–1.09)	< 0.001	1.09 (1.09–1.09)	< 0.001	1.09 (1.08–1.09)	< 0.001	1.09 (1.08–1.09)	< 0.001
Male	1.46 (1.37–1.55)	< 0.001	1.43 (1.35–1.52)	< 0.001	1.46 (1.37–1.55)	< 0.001	1.28 (1.20–1.37)	< 0.001
Race								
Black	Reference		Reference		Reference		Reference	
Hispanic	0.69 (0.63–0.75)	< 0.001	0.73 (0.67–0.80)	< 0.001	0.70 (0.64–0.76)	< 0.001	0.74 (0.67–0.80)	< 0.001
White	0.81 (0.75–0.87)	< 0.001	0.79 (0.74–0.86)	< 0.001	0.82 (0.76–0.88)	0.002	0.83 (0.78–0.90)	< 0.001
Others	0.55 (0.45–0.66)	< 0.001	0.55 (0.46–0.67)	< 0.001	0.55 (0.46–0.66)	< 0.001	0.61 (0.50–0.73)	< 0.001
Obesity								
Normal	Reference		Reference		Reference		Reference	
Obese	0.87 (0.86–0.93)	< 0.001	0.90 (0.84–0.97)	0.005	0.87 (0.81–0.94)	< 0.001	0.86 (0.80–0.93)	<0.001
Overweight	1.03 (0.13–1.12)	0.409	1.10 (1.02–1.19)	0.014	1.03 (0.95–1.10)	0.503	0.99 (0.91–1.07)	0.767
Advanced fibrosis			1.33 (1.21–1.46)	< 0.001	1.21 (1.10–1.33)	< 0.001	1.22 (1.11–1.35)	< 0.001
T2DM					2.00 (1.83–2.18)	< 0.001	1.94 (1.77–2.11)	< 0.001
HTN							1.30 (1.22–1.39)	< 0.001
HL							0.92 (0.85–0.98)	0.017
Smoking							1.58 (1.48–1.69)	< 0.001

Model 1 was adjusted for age, sex, race, and BMI levels. Model 2 was adjusted for age, sex, race, BMI levels, and the existence of advanced fibrosis. Model 3 was adjusted for age, sex, race, BMI levels, advanced fibrosis, and T2DM. Model 4 was adjusted for age, sex, race, BMI levels, advanced fibrosis, T2DM, HTN, hypercholesterolemia, and history of smoking.

MAFLD, metabolic dysfunction-associated fatty liver diseases; BMI, body mass index; T2DM, Type 2 Diabetes; HL, hyperlipidemia; HTN, Hypertension.

For the purpose of modifying the interaction between age and sarcopenia, adjusted HRs calculated for individuals with sarcopenia were split into two age groups (**Table 4**). Higher levels of activity improved the survival of sarcopenia (Insufficiently active, HR = 0.75 [95% CI 0.58–0.97]; Active, HR=0.64 [95% CI 0.48–0.86), which was more prominent in older patients. In both age categories, adequate protein intake was not significantly associated with long-term outcomes.

**Table 4:** Hazard Ratios of Risk Factors of Patients with Sarcopenia for All-cause Mortality, Stratified by Age.

Covariate	Overall		< 60 years		≥ 60 years	
	HR (95% CI)	P-value	HR (95% CI)	P-value	HR (95% CI)	P-value
Age	1.07 (1.06–1.08)	< 0.001	1.07 (1.05–1.09)	< 0.001	1.10 (1.06–1.13)	< 0.001
Male	1.47 (1.15–1.88)	0.002	1.92 (1.28–2.88)	0.002	1.21 (0.87–1.67)	0.251
Race						
Black	Reference		Reference		Reference	
Hispanic	0.87 (0.65–1.17)	0.353	0.87 (0.53–1.43)	0.581	0.85 (0.58–1.23)	0.383
White	0.98 (0.76–1.27)	0.896	0.78 (0.50–1.22)	0.276	1.14 (0.83–1.56)	0.435
Others	1.06 (0.46–2.48)	0.884	1.20 (0.35-4.08)	0.768	0.92 (0.28–3.08)	0.896
Physical activity						
Inactive	Reference		Reference		Reference	
Insufficiently active	0.75 (0.58–0.97)	0.027	0.74 (0.45–1.02)	0.176	0.78 (0.57–1.08)	0.132
Active	0.64 (0.48–0.86)	0.003	0.80 (0.51–1.30)	0.392	0.60 (0.42–0.86)	0.006
Relative protein intake						
< 1.2 g/kg	Reference		Reference		Reference	
1.2–1.5 g/kg	0.93 (0.43–1.99)	0.851	0.72 (0.26–2.00)	0.534	1.09 (0.34–3.51)	0.886
> 1.5g/kg	0.90 (0.56–1.45)	0.667	1.14 (0.57–2.29)	0.715	0.77 (0.40–1.49)	0.434
Cirrhosis	1.12 (0.86–1.45)	0.395	0.96 (0.60–1.56)	0.883	1.15 (0.84–1.59)	0.383
T2DM	1.39 (1.05–1.82)	0.02	1.76 (1.08–2.85)	0.023	1.26 (0.90–1.78)	0.182
HTN	1.12 (0.90–1.40)	0.311	1.31 (0.90–1.91)	0.165	1.00 (0.76–1.32)	0.983
HL	1.02 (0.80–1.31)	0.862	0.82 (0.52–1.31)	0.409	1.08 (0.79–1.47)	0.64
Smoking	1.40 (1.11–1.77)	0.004	1.36 (0.92–2.01)	0.123	1.54 (1.14–2.07)	0.005

Adjusted for age, sex, and race.

T2DM, Type 2 Diabetes; HL, hyperlipidemia; HTN, Hypertension.

Diabetes had the greatest impact on the prognosis of persons with MAFLD (HR = 1.84 [95% CI 1.59–2.12]), and increasing activity levels also improved the survival (Insufficiently active, HR = 0.85 [95% CI 0.73–0.99]; Active, HR = 0.64 [95% CI 0.67–0.93]). A daily protein intake of greater than 1.5 g/kg protein was associated with a better prognosis in older MAFLD patients, but had no significant effect on younger individuals (**Table S8**).

## 4 Discussion

In this study, we used data sets from NHANES III (1988–1994) to investigate the clinical impact of dietary components and physical activity on patients with sarcopenia and MAFLD, revealing an increased incidence of sarcopenia in patients with MAFLD. Decreased physical activity levels and insufficient protein consumption may contribute to sarcopenia, with reduced physical activity being related to unfavorable outcomes.

Sarcopenia is strongly age-related and primarily observed in older people, while chronic diseases may induce sarcopenia in younger individuals (42). Consistent with earlier studies that demonstrated a positive correlation between NAFLD and sarcopenia (43, 44), sarcopenia was more prevalent in MAFLD than non-MAFLD participants (11.7% vs. 3.0%) in our study and related to a higher mortality. Insulin resistance may function as the main pathologic mechanism of MAFLD and sarcopenia. Insulin could activate the mammalian target of rapamycin (mTOR) and enhance its downstream effectors, 4E-binding protein 1 and ribosomal S6 kinase 1, mediating skeletal muscle anabolism and maintaining muscle mass (45). Impaired insulin sensitivity may interrupt the glucose metabolism and result in excess glucose conversion to triacylglycerol in the liver, which also leads to hepatic insulin resistance. Other factors, such as chronic inflammation, hyperammonemia, alterations in sex hormones, and insulin-like growth factor-1 signaling may also interfere with the glucose disposal in skeletal muscles and lead to muscle loss (42, 46, 47), which helps to explain the co-existence of sarcopenia with MAFLD. The impact of sarcopenia on the long-term prognosis of MAFLD is anticipated to be substantial, since both sarcopenia and liver fibrosis caused by MAFLD are independently associated with increased risk of death from all causes (47).

Prior studies showed a strong interest in elucidating how sarcopenia contributes to adverse outcomes in patients with chronic liver diseases, particularly those with cirrhosis. Molecular studies have shown that cirrhotic patients had an increase in muscle cell autophagy (48) and a higher expression of myostatin that inhibited mTOR signaling and suppressed protein synthesis (49). Besides, hyperammonia, a common abnormality caused by liver dysfunction and portosystemic shunting, may contribute to both myostatin upregulation and autophagy processes (48–50). In addition to the detrimental impact of sarcopenia on cirrhosis, additional investigation is needed to understand how sarcopenia affects the prognosis of MAFLD. Moreover, we confirmed that sarcopenia was an independent predictor of survival in individuals either with or without MAFLD. Our research further revealed a strong correlation between MAFLD and T2DM rather than severe fibrosis, indicating that metabolic dysregulation was mainly responsible for the unfavorable prognosis of MAFLD patients in the general community.

Despite compelling evidence that sarcopenia is associated with negative outcomes, no viable methods to reverse muscle mass loss have been identified (51). Previous studies supported the hypothesis that physical activity can enhance the functional capacity of skeletal muscle, but its effect on gaining muscle mass remained uncertain (52). Here, we revealed that in patients diagnosed with sarcopenia, increasing the intensity and frequency of exercises is linked to a better prognosis, especially in the older population. Exercise may boost the muscle accumulation by increasing hormone levels such as testosterone (53) and insulin-like growth factor-1 (IGF-1) (54), and it may promote mitochondrial biogenesis by inhibiting TNF-α and various other molecular mechanisms. Exercise also upregulated PGC-1α and Toll-like receptors downregulation that enhanced the anti-inflammatory and anti-atrophy effects (55, 56). Autophagy contributes to decreased synthesis and increased proteolysis of skeletal muscle in patients with chronic liver diseases. Physical exercise may rescue the impaired mTORC1 signaling by stimulating phosphatidic acid (57), therefore maintaining muscle mass by activating protein synthesis and inhibiting autophagy. As resistance exercises were more effective at stimulating skeletal muscle protein synthesis (58), the effect of different types of exercise on preventing sarcopenia and improving survival required more validation.

Given that physical activity was rather compromised in older people by their frailty or diseases, a protein supplement was considered a practical choice for preserving muscle mass (59, 60). Although older and younger individuals had similar rates of protein turnover (61), elderly people have a more muted response to administered amino acids than young people (62). Lower mTOR and p70S6K concentrations (63), along with a concurrent decline in positive regulators (such as IGF-1) and an increase in negative regulators (such as AMPK) in older skeletal muscle, may explain their resistance to amino acid feedings (64). Some observational and cohort studies demonstrate that adequate protein consumption is well tolerated without major adverse events and can prevent muscle loss (65, 66), but there is insufficient evidence to support the hypothesis that protein intake can improve the long-term outcomes. Several randomized controlled trials were performed in cirrhotic individuals with sarcopenia; nevertheless, nutrition supplementation through multiple routes had little influence on sarcopenia or survival (30). Supplemental hormone therapy and mechanistic targeted treatments were produced as more precise treatments for sarcopenia, necessitating a clearer knowledge for its pathophysiological process (51, 67).

This research has a few limitations. First, the diagnosis of FLD was established by ultrasound images from NHANES III, but fibrosis data were not available with ultrasound. In the absence of liver stiffness measurement (LSM) results, advanced fibrosis was determined by NFS and FIB-4 scores. The relationship between MAFLD-related fibrosis and sarcopenia should be evaluated further. Moreover, the NHANES III database was relatively outdated in comparison to other NHANES survey cycles. Second, we calculated the skeletal muscle mass using BIA measurements, whereas dual-energy X-ray absorptiometry (DXA) is the primary method for measuring body composition. Since sarcopenia is defined as loss of both muscle mass and function, the NHANES database does not contain muscle function measurements, such as contractile strength, maintenance of contraction, and muscle fatigue in response to persistent and repetitive contraction (68). Finally, the mortality data came from a separate national database that matched the NHANES III data, where the data on liver-associated mortality was not available. Given the cross-sectional nature of the NHANES database, the progression of liver diseases cannot be determined.

In summary, our data demonstrate that sarcopenia is more prevalent and is associated with an increased risk of all-cause death among MAFLD participants. MAFLD patients who suffer from sarcopenia may benefit from physical activity and a proper intake of proteins. Therefore, clinicians should recognize and manage sarcopenia in patients with MAFLD in order to improve their life quality and overall survival outcome.

## Data availability statement

The original contributions presented in the study are included in the article/**Supplementary Material**. Further inquiries can be directed to the corresponding author.

## Ethics statement

The studies involving human participants were reviewed and approved by NCHS Ethics Review Board. The patients/participants provided their written informed consent to participate in this study.

## Author contributions

YY and YC: conception and design. YY, CW, YD, JH, and YL: data collection, data management, and formal statistical analysis. YY and CW: manuscript writing. YC: manuscript revising. All authors involved in writing and approved the final manuscript.
