# Supplementary material for: Diet was less significant than physical activity in the prognosis of people with sarcopenia and metabolic dysfunction-associated fatty liver diseases: Analysis of the National Health and Nutrition Examination Survey III
Source: Front Endocrinol (Lausanne). 2023 Feb 23;14:1101892. doi: 10.3389/fendo.2023.1101892 (PMC9995978; doi:10.3389/fendo.2023.1101892)
Supplement: Supplementary file 1 [file DataSheet_1.docx]

**Table S1 Demographic, Dietary, and Physical Activity Characteristics of Participants Categorized by the Presence of MAFLD and Sarcopenia**

| Covariate | MAFLD | | | Non-MAFLD | | |
| --- | --- | --- | --- | --- | --- | --- |
|  |  |  |  |  |  |  |
|  | Sarcopenia | Non-sarcopenia | P-value | Sarcopenia | Non-sarcopenia | P-value |
|  | N=290 ^a^ | N=2183 ^a^ |  | N=295 ^a^ | N=9491 ^a^ |  |
| Male (%) | 114 (39.3) | 1184 (54.2) | <0.001 | 102 (34.6) | 4462 (47.0) | <0.001 |
| Race (%) |  |  |  |  |  |  |
| Black | 77 (26.6) | 411 (18.8) | <0.001 | 132 (44.7) | 2861 (30.1) | <0.001 |
| Hispanic | 78 (26.9) | 913 (41.8) |  | 69 (23.4) | 2549 (26.9) |  |
| Others | 7 (2.4) | 85 (3.9) |  | 7 (2.4) | 418 (4.4) |  |
| White | 128 (44.1) | 774 (35.5) |  | 87 (29.5) | 3663 (38.6) |  |
| Age (years) | 53.64 (14.31) | 48.93 (14.63) | <0.001 | 53.47 (15.66) | 42.01 (15.81) | <0.001 |
| Weight (kg) | 108.71 (24.05) | 84.29 (15.91) | <0.001 | 102.45 (23.94) | 72.36 (15.12) | <0.001 |
| Height (cm) | 166.31 (10.74) | 166.49 (9.70) | 0.772 | 165.10 (10.79) | 176.50 (910.74) | 0.83 |
| BMI (kg/m^2^) | 39.20 (7.35) | 30.36 (4.91) | <0.001 | 37.47 (7.36) | 25.85 (4.80) | <0.001 |
| Waist (cm) | 121.43 (13.47) | 102.42 (11.20) | <0.001 | 115.43 (13.99) | 89.23 (12.58) | <0.001 |
| Calorie (kcal) ^b^ | 1734.00 [1220.75, 2288.50] | 1953.50 [1402.25, 2654.50] | <0.001 | 1604.00 [1164.75, 2286.00] | 1967.00 [1431.00, 2652.50] | <0.001 |
| Carbohydrates (g) ^b^ | 208.05 [143.75, 289.08] | 235.05 [170.22, 316.55] | <0.001 | 210.80 [141.40, 268.40] | 239.30 [173.00, 323.20] | <0.001 |
| Protein (g) ^b^ | 65.85 [49.83, 92.18] | 75.30 [51.82, 102.47] | 0.002 | 64.15 [44.50, 88.95] | 73.60 [51.50, 101.90] | <0.001 |
| Fat (g) ^b^ | 63.20 [39.20, 91.00] | 70.10 [46.70, 102.65] | 0.003 | 58.25 [36.50, 93.20] | 72.00 [47.10, 106.25] | <0.001 |
| Saturated fat (g) ^b^ | 19.95 [12.60, 30.70] | 22.50 [14.50, 33.70] | 0.013 | 18.85 [11.00, 29.80] | 23.30 [14.80, 35.10] | <0.001 |
| Monounsaturated fat (g) ^b^ | 24.20 [14.60, 34.58] | 26.20 [17.10, 39.68] | 0.003 | 21.85 [13.40, 33.95] | 26.70 [17.10, 40.00] | <0.001 |
| Polyunsaturated fat (g) ^b^ | 12.30 [7.00, 20.12] | 14.20 [8.30, 22.00] | 0.01 | 11.85 [6.97, 20.12] | 14.30 [8.50, 23.20] | 0.001 |
| Cholesterol (mg) ^b^ | 218.50 [128.25, 388.25] | 254.00 [139.00, 440.75] | 0.024 | 190.50 [112.75, 315.00] | 229.00 [131.00, 404.00] | <0.001 |
| Percentage of energy from |  |  |  |  |  |  |
| Carbohydrates (%) | 49.44 (12.37) | 49.39 (11.17) | 0.94 | 50.37 (12.35) | 49.64 (11.40) | 0.293 |
| Protein (%) | 16.65 (5.98) | 16.00 (4.90) | 0.041 | 15.96 (4.85) | 15.55 (5.01) | 0.169 |
| Fat (%) | 33.56 (10.24) | 33.27 (9.29) | 0.624 | 33.49 (10.27) | 33.45 (9.42) | 0.944 |
| Physical activity (%) |  |  |  |  |  |  |
| Inactive | 94 (32.4) | 513 (23.5) | <0.001 | 95 (32.2) | 1723 (18.2) | <0.001 |
| Insufficient | 131 (45.2) | 972 (44.5) |  | 110 (37.3) | 3927 (41.4) |  |
| Active | 65 (22.4) | 698 (32.0) |  | 90 (30.5) | 3841 (40.5) |  |
| Protein intake (%) |  |  |  |  |  |  |
| <1.2g/kg | 261 (91.3) | 1560 (73.2) | <0.001 | 260 (91.5) | 5662 (61.4) | <0.001 |
| 1.2-1.5g/kg | 10 (3.5) | 298 (14.0) |  | 6 (2.1) | 2106 (22.8) |  |
| >1.5g/kg | 15 (5.2) | 272 (12.8) |  | 18 (6.3) | 1459 (15.8) |  |
| Carbohydrate level (%) |  |  |  |  |  |  |
| <40% | 81 (28.3) | 382 (17.9) | <0.001 | 82 (28.9) | 1636 (17.7) | <0.001 |
| ≥40% | 205 (71.7) | 1748 (82.1) |  | 202 (71.1) | 7591 (82.3) |  |
| Mortality (%) | 181 (62.4) | 983 (45.1) | <0.001 | 182 (61.7) | 2925 (30.8) | <0.001 |
| Causes of death (%) |  |  |  |  |  |  |
| Diabetes | 18 (6.2) | 55 (2.5) | <0.001 | 6 (2.0) | 86 (0.9) | <0.001 |
| Heart | 50 (17.2) | 283 (13.0) |  | 58 (19.7) | 792 (8.3) |  |
| Malignancy | 34 (11.7) | 221 (10.1) |  | 33 (11.2) | 753 (7.9) |  |
| Others | 188 (64.8) | 1624 (74.4) |  | 198 (67.1) | 7860 (82.8) |  |
| Diabetes (%) | 57 (19.7) | 338 (15.5) | 0.082 | 36 (12.2) | 466 (4.9) | <0.001 |
| Hypertension (%) | 149 (51.4) | 779 (35.7) | <0.001 | 148 (50.2) | 1969 (20.7) | <0.001 |
| Cardiovascular diseases (%) | 20 (6.9) | 106 (4.9) | 0.179 | 12 (4.1) | 257 (2.7) | 0.22 |
| Chronic kidney diseases (%) | 14 (4.8) | 98 (4.5) | 0.912 | 25 (8.5) | 326 (3.4) | < 0.001 |
| Cancer (%) | 18 (6.2) | 123 (5.6) | 0.795 | 25 (8.5) | 479 (5.0) | 0.013 |
| Hyperlipidemia (%) | 66 (22.8) | 482 (22.1) | 0.852 | 58 (19.7) | 1529 (16.1) | 0.121 |
| Smoking (%) | 155 (53.4) | 1160 (53.1) | 0.971 | 138 (46.8) | 4809 (50.7) | 0.209 |

Abbreviations: BMI, body mass index; MAFLD, metabolic (dysfunction)-associated fatty liver diseases.

^a^ The number of individuals was reported by multiplying the estimated percentage by the total number of individuals in the full sample.

^b^ Median (interquartile range).

**Table S2** **Laboratory Characteristics of Participants Categorized by the Presence of MAFLD and Sarcopenia**

| Covariate | MAFLD+ | | | MAFLD- | | | MAFLD+ | | | | | |
| --- | --- | --- | --- | --- | --- | --- | --- | --- | --- | --- | --- | --- |
|  |  |  |  |  |  |  | < 60 years | | | >= 60 years | | |
|  | Sarcopenia | Non-sarcopenia | P-value | Sarcopenia | Non-sarcopenia | P-value | Sarcopenia | Non-sarcopenia | P-value | Sarcopenia | Non-sarcopenia | P-value |
|  | N=290^a^ | N=2183 ^a^ |  | N=348 ^a^ | N=10633 ^a^ |  | N=164 ^a^ | N=1511 ^a^ |  | N=126 ^a^ | N=672 ^a^ |  |
| ALT (U/L) ^b^ | 73.44 (204.36) | 54.94 (156.73) | 0.07 | 103.16 (262.99) | 68.96 (208.23) | 0.003 | 88.09 (226.29) | 61.45 (164.20) | 0.059 | 54.37 (170.69) | 40.25 (137.36) | 0.31 |
| AST (U/L) ^b^ | 75.88 (203.56) | 54.99 (156.28) | 0.04 | 106.87 (261.70) | 73.31 (207.13) | 0.003 | 88.77 (225.75) | 59.43 (164.06) | 0.037 | 59.09 (169.82) | 44.99 (136.71) | 0.308 |
| Albumin (g/L) ^b^ | 40.06 (3.36) | 41.58 (3.50) | <0.001 | 39.89 (3.64) | 41.88 (3.64) | <0.001 | 39.92 (3.27) | 41.81 (3.60) | <0.001 | 40.24 (3.47) | 41.09 (3.22) | 0.009 |
| Creatinine (μmol/L) ^b^ | 92.16 (18.30) | 95.08 (21.70) | 0.034 | 93.85 (21.10) | 94.40 (30.08) | 0.747 | 89.16 (15.88) | 92.20 (17.35) | 0.038 | 95.93 (20.40) | 101.50 (28.12) | 0.038 |
| GGT (U/L) ^b^ | 48.60 (53.25) | 48.32 (67.57) | 0.954 | 34.72 (28.68) | 29.11 (41.85) | 0.033 | 45.27 (50.68) | 51.82 (71.10) | 0.332 | 52.57 (56.17) | 40.45 (58.16) | 0.058 |
| Total bilirubin (mg/dL) ^b^ | 0.55 (0.36) | 0.60 (0.32) | 0.016 | 0.52 (0.27) | 0.60 (0.34) | <0.001 | 0.49 (0.20) | 0.61 (0.34) | <0.001 | 0.62 (0.49) | 0.59 (0.26) | 0.228 |
| Cholesterol (mg/dL) ^b^ | 221.09 (46.66) | 220.60 (44.97) | 0.867 | 225.06 (45.19) | 203.71 (44.10) | <0.001 | 210.99 (43.50) | 217.28 (45.19) | 0.101 | 233.78 (47.56) | 228.00 (43.59) | 0.188 |
| Triglyceride (mg/dL) ^b^ | 181.73 (132.56) | 210.20 (174.93) | 0.023 | 159.44 (117.90) | 122.11 (111.19) | <0.001 | 176.65 (154.18) | 209.31 (179.17) | 0.06 | 187.87 (100.85) | 212.22 (165.10) | 0.166 |
| HDL-C (mg/dl) ^b^ | 45.57 (12.39) | 44.42 (13.42) | 0.177 | 48.46 (13.22) | 52.13 (15.31) | <0.001 | 42.64 (10.71) | 43.98 (12.70) | 0.205 | 49.28 (13.38) | 45.39 (14.87) | 0.007 |
| Platelet (10^9^/L) ^b^ | 285.90 (73.24) | 278.05 (72.51) | 0.091 | 297.12 (81.36) | 276.01 (69.86) | <0.001 | 297.18 (73.69) | 286.25 (72.68) | 0.077 | 271.78 (70.46) | 259.59 (68.69) | 0.073 |
| Fasting glucose (mg/dL) ^b^ | 122.00 (64.86) | 117.50 (56.04) | 0.214 | 115.21 (55.76) | 97.12 (29.10) | <0.001 | 123.46 (76.35) | 113.25 (53.32) | 0.03 | 120.19 (46.96) | 126.97 (60.64) | 0.236 |
| Fasting insulin (uIU/L) ^b^ | 24.08 (17.82) | 18.59 (17.89) | <0.001 | 18.84 (22.16) | 10.59 (12.02) | <0.001 | 26.05 (20.56) | 18.42 (17.77) | <0.001 | 21.61 (13.28) | 18.96 (18.14) | 0.124 |
| CRP (mg/L) ^b^ | 0.90 (1.16) | 0.51 (0.69) | <0.001 | 0.97 (1.69) | 0.42 (0.70) | <0.001 | 0.97 (1.11) | 0.50 (0.67) | <0.001 | 0.82 (1.22) | 0.53 (0.73) | <0.001 |
| HbA1c (%) | 6.19 (1.55) | 6.02 (1.59) | 0.109 | 6.06 (1.54) | 5.37 (0.91) | <0.001 | 6.23 (1.67) | 5.89 (1.59) | 0.011 | 6.13 (1.39) | 6.32 (1.57) | 0.204 |
| HOMA-IR ^b^ | 7.71 (8.35) | 5.96 (9.31) | 0.003 | 6.45 (19.57) | 2.77 (4.96) | <0.001 | 8.40 (9.24) | 5.53 (8.00) | <0.001 | 6.86 (7.02) | 6.92 (11.66) | 0.95 |
| NFS ^b^ | -0.69 (1.47) | -1.91 (1.53) | <0.001 | -0.54 (1.62) | -2.22 (1.72) | <0.001 | -1.01 (1.45) | -2.42 (1.35) | <0.001 | -0.29 (1.41) | -0.76 (1.28) | <0.001 |
| FIB-4 ^b^ | 1.27 (1.24) | 1.13 (1.75) | 0.2 | 1.66 (1.58) | 1.17 (1.40) | <0.001 | 0.94 (0.76) | 0.85 (0.65) | 0.125 | 1.68 (1.56) | 1.75 (2.91) | 0.791 |
| Advanced fibrosis (%) | 51 (17.6) | 156 (7.1) | <0.001 | 142 (27.4) | 1106 (9.2) | <0.001 | 24 (14.6) | 62 (4.1) | <0.001 | 27 (21.4) | 94 (14.0) | 0.045 |

Abbreviations: ALT, alanine aminotransferase; AST, aspartate aminotransferase; GGT, gamma-glutamyl transferase; HDL-C, high-density lipoprotein cholesterol; HbA1c, glycated hemoglobin; CRP, C-reactive protein; HOMA-IR, homeostasis model assessment of insulin resistance score, NFS, Non-alcoholic fatty liver diseases (NAFLD) fibrosis score; FIB-4, Fibrosis-4 index; MAFLD, metabolic (dysfunction)-associated fatty liver diseases.

^a^ The number of individuals was reported by multiplying the estimated percentage by the total number of individuals in the full sample.

^b^ Mean (Standard deviation).

**Table S3** **Prevalence of Sarcopenia in MAFLD and Non-MAFLD Participants**

|  | MAFLD | | Non-MAFLD | | P-value |
| --- | --- | --- | --- | --- | --- |
|  | N | n (%) | N | n (%) |  |
| Total | 2473 | 290 (11.7) | 9786 | 295 (3.0) | <0.001 |
| Age, <60 | 1645 | 164 (9.8) | 7737 | 158 (2.0) | <0.001 |
| Age, ≥60 | 798 | 126 (15.8) | 2049 | 137 (6.7) | <0.001 |
| Male | 1298 | 114 (8.8) | 4564 | 102 (2.2) | <0.001 |
| Female | 1175 | 176 (15.0) | 5222 | 193 (3.7) | <0.001 |
| Race, black | 488 | 77 (15.8) | 2993 | 132 (4.4) | <0.001 |
| Race, Hispanic | 991 | 78 (7.9) | 2618 | 69 (2.6) | <0.001 |
| Race, white | 902 | 128 (14.2) | 3750 | 87 (2.3) | <0.001 |
| Race, others | 92 | 7 (7.6) | 425 | 7 (1.6) | <0.001 |
| Physical activity, active | 763 | 65 (8.5) | 3931 | 90 (2.3) | <0.001 |
| Physical activity, insufficient | 1103 | 131 (11.9) | 4037 | 110 (2.7) | <0.001 |
| Physical activity, inactive | 607 | 94 (15.5) | 1818 | 95 (5.2) | <0.001 |
| Calorie intake, Q1 ^a^ | 652 | 101 (15.5) | 2330 | 99 (4.2) | <0.001 |
| Calorie intake, Q2 ^a^ | 629 | 57 (9.1) | 2344 | 78 (3.3) | <0.001 |
| Calorie intake, Q3 ^a^ | 597 | 69 (11.6) | 2388 | 59 (2.5) | <0.001 |
| Calorie intake, Q4 ^a^ | 538 | 59 (11.0) | 2449 | 48 (2.0) | <0.001 |
| Normal or mild fibrosis | 2266 | 239 (10.5) | 9178 | 233 (2.5) | <0.001 |
| Advanced fibrosis | 207 | 51 (24.6) | 608 | 62 (10.2) | <0.001 |

Abbreviations: MAFLD, metabolic (dysfunction)-associated fatty liver diseases.

^a^ Q1: 0–1763 kcal in male, 0–1230 kcal in female; Q2: 1764–2365 kcal in male, 1231–1647 kcal in female; Q3: 2366–3128 kcal in male, 1648–2148 kcal in female; Q4: >3128 kcal in male, >2148 kcal in female.

**Table S4** **Ordinal Logistic Regression for Improved Physical Activity Levels, Stratified by the presence of MAFLD**

| Covariate | Overall | | MAFLD | | Non-MAFLD | |
| --- | --- | --- | --- | --- | --- | --- |
|  | OR (95% CI) | P-value | OR (95% CI) | P-value | OR (95% CI) | P-value |
| Sarcopenia | 0.74 (0.62-0.87) | <0.001 | 0.76 (0.6-0.97) | 0.03 | 0.75 (0.6-0.95) | 0.018 |
| Age | 0.99 (0.99-1) | <0.001 | 1 (0.99-1) | 0.227 | 0.99 (0.99-1) | <0.001 |
| Male | 1.82 (1.69-1.95) | <0.001 | 1.86 (1.58-2.18) | <0.001 | 1.84 (1.7-1.99) | <0.001 |
| Race |  |  |  |  |  |  |
| Black | Reference |  | Reference |  | Reference |  |
| Hispanic | 0.65 (0.59-0.71) | <0.001 | 0.62 (0.5-0.76) | <0.001 | 0.67 (0.61-0.74) | <0.001 |
| White | 1.34 (1.23-1.46) | <0.001 | 1.24 (1.01-1.54) | 0.044 | 1.39 (1.26-1.52) | <0.001 |
| Others | 0.83 (0.69-0.99) | 0.04 | 0.77 (0.5-1.17) | 0.221 | 0.86 (0.71-1.05) | 0.133 |
| Obesity |  |  |  |  |  |  |
| Normal | Reference |  | Reference |  | Reference |  |
| Overweight | 0.94 (0.87-1.02) | 0.146 | 1.22 (0.91-1.63) | 0.193 | 0.94 (0.87-1.03) | 0.194 |
| Obese | 0.73 (0.66-0.79) | <0.001 | 0.83 (0.62-1.11) | 0.217 | 0.8 (0.72-0.89) | <0.001 |
| Diabetes | 0.78 (0.68-0.89) | <0.001 | 0.92 (0.75-1.14) | 0.466 | 0.73 (0.61-0.87) | 0.001 |
| Hypertension | 0.91 (0.84-0.99) | 0.032 | 0.9 (0.77-1.06) | 0.221 | 0.93 (0.84-1.02) | 0.134 |
| Hypercholesterolemia | 1.19 (1.08-1.3) | <0.001 | 1.16 (0.97-1.4) | 0.113 | 1.2 (1.08-1.33) | 0.001 |
| Smoking | 0.86 (0.8-0.92) | <0.001 | 0.93 (0.8-1.09) | 0.375 | 0.84 (0.77-0.91) | <0.001 |
| Advanced fibrosis | 0.83 (0.72-0.95) | 0.009 | 0.74 (0.55-0.99) | 0.04 | 0.85 (0.72-0.99) | 0.042 |

Abbreviations: OR, Odds ratio; CI, Confidence interval; MAFLD, metabolic (dysfunction)-associated fatty liver diseases.

Physical Activity Levels defined as (1=Inactive, 2=Insufficiently active, 3=Active)

**Table S5** **Ordinal Logistic Regression for Improved Protein Intake Levels, Stratified by the presence of MAFLD**

| Covariate | Overall | | MAFLD | | Non-MAFLD | |
| --- | --- | --- | --- | --- | --- | --- |
|  | OR (95% CI) | P-value | OR (95% CI) | P-value | OR (95% CI) | P-value |
| Sarcopenia | 0.48 (0.35-0.65) | <0.001 | 0.51 (0.32-0.78) | 0.003 | 0.49 (0.31-0.74) | 0.001 |
| Age | 0.98 (0.98-0.99) | <0.001 | 0.98 (0.97-0.98) | <0.001 | 0.99 (0.98-0.99) | <0.001 |
| Male | 1.87 (1.72-2.02) | <0.001 | 2.08 (1.68-2.58) | <0.001 | 1.83 (1.67-1.99) | <0.001 |
| Race |  |  |  |  |  |  |
| Black | Reference |  | Reference |  | Reference |  |
| Hispanic | 1.47 (1.33-1.63) | <0.001 | 1.56 (1.18-2.07) | 0.002 | 1.45 (1.3-1.62) | <0.001 |
| White | 1.15 (1.04-1.28) | 0.005 | 1.21 (0.9-1.64) | 0.216 | 1.15 (1.03-1.28) | 0.011 |
| Others | 1.49 (1.23-1.81) | <0.001 | 2.12 (1.26-3.51) | 0.004 | 1.4 (1.14-1.73) | 0.001 |
| Obesity |  |  |  |  |  |  |
| Normal | Reference |  | Reference |  | Reference |  |
| Overweight | 0.51 (0.47-0.56) | <0.001 | 0.46 (0.34-0.62) | <0.001 | 0.52 (0.47-0.57) | <0.001 |
| Obese | 0.25 (0.22-0.28) | <0.001 | 0.26 (0.19-0.35) | <0.001 | 0.23 (0.2-0.27) | <0.001 |
| Diabetes | 0.91 (0.76-1.08) | 0.278 | 0.96 (0.71-1.29) | 0.782 | 0.93 (0.74-1.15) | 0.496 |
| Hypertension | 0.98 (0.88-1.09) | 0.678 | 0.91 (0.73-1.14) | 0.426 | 1.01 (0.89-1.14) | 0.889 |
| Hypercholesterolemia | 1.06 (0.95-1.19) | 0.282 | 1.07 (0.84-1.37) | 0.578 | 1.05 (0.92-1.19) | 0.46 |
| Smoking | 1.01 (0.94-1.1) | 0.72 | 1.12 (0.92-1.38) | 0.265 | 0.99 (0.91-1.08) | 0.81 |
| Advanced fibrosis | 0.87 (0.73-1.04) | 0.125 | 0.63 (0.4-0.98) | 0.046 | 0.93 (0.76-1.13) | 0.457 |

Abbreviations: OR, Odds ratio; CI, Confidence interval; MAFLD, metabolic (dysfunction)-associated fatty liver diseases.

Protein Intake Levels defined as (1=”<1.2 g/kg”, 2=”1.2-1.5g/kg”, 3=”>1.5g/kg”)

**Table S6** **Ordinal Logistic Regression for Improved** **Calorie and Carbohydrates Intake Levels (in Quartiles), Stratified by the presence of MAFLD**

| Covariate | Calorie | | | | Carbohydrates | | | |
| --- | --- | --- | --- | --- | --- | --- | --- | --- |
|  | MAFLD | | Non-MAFLD | | MAFLD | | Non-MAFLD | |
|  | OR (95% CI) | P-value | OR (95% CI) | P-value | OR (95% CI) | P-value | OR (95% CI) | P-value |
| Sarcopenia | 0.91 (0.72-1.16) | 0.4610 | 0.9 (0.71-1.12) | 0.3370 | 0.84 (0.66-1.07) | 0.1530 | 0.95 (0.76-1.18) | 0.6320 |
| Age | 0.96 (0.96-0.97) | <0.001 | 0.97 (0.97-0.97) | <0.001 | 0.97 (0.96-0.97) | <0.001 | 0.97 (0.97-0.98) | <0.001 |
| Male | 0.89 (0.76-1.04) | 0.1390 | 0.99 (0.92-1.07) | 0.7600 | 0.86 (0.74-1.01) | 0.0650 | 1.03 (0.96-1.11) | 0.4310 |
| Race |  |  |  |  |  |  |  |  |
| Black | Reference |  | Reference |  | Reference |  | Reference |  |
| Hispanic | 1.15 (0.93-1.41) | 0.1930 | 1.02 (0.93-1.13) | 0.6450 | 1.4 (1.14-1.71) | 0.0010 | 1.16 (1.06-1.28) | 0.0020 |
| White | 1.54 (1.25-1.9) | <0.001 | 1.38 (1.26-1.51) | <0.001 | 1.57 (1.28-1.94) | <0.001 | 1.43 (1.31-1.57) | <0.001 |
| Others | 1.12 (0.75-1.68) | 0.5830 | 0.85 (0.7-1.02) | 0.0820 | 1.61 (1.06-2.43) | 0.0250 | 1.17 (0.97-1.41) | 0.1050 |
| Obesity |  |  |  |  |  |  |  |  |
| Normal | Reference |  | Reference |  | Reference |  | Reference |  |
| Overweight | 0.73 (0.56-0.97) | 0.0310 | 0.88 (0.81-0.96) | 0.0040 | 0.82 (0.62-1.09) | 0.1720 | 0.89 (0.82-0.97) | 0.0070 |
| Obese | 0.84 (0.63-1.11) | 0.2100 | 0.96 (0.86-1.06) | 0.4230 | 0.92 (0.7-1.22) | 0.5770 | 0.87 (0.78-0.97) | 0.0090 |
| Diabetes | 0.73 (0.59-0.9) | 0.0030 | 0.62 (0.52-0.74) | <0.001 | 0.58 (0.47-0.72) | <0.001 | 0.56 (0.47-0.67) | <0.001 |
| Hypertension | 0.97 (0.83-1.14) | 0.7490 | 0.94 (0.86-1.04) | 0.2300 | 0.88 (0.75-1.03) | 0.1210 | 0.99 (0.9-1.09) | 0.8540 |
| Hypercholesterolemia | 1.14 (0.95-1.37) | 0.1510 | 1.04 (0.94-1.15) | 0.4680 | 1.26 (1.05-1.51) | 0.0130 | 1.13 (1.02-1.25) | 0.0220 |
| Smoking | 1.18 (1.01-1.38) | 0.0320 | 1.07 (0.99-1.15) | 0.0950 | 1.09 (0.94-1.27) | 0.2640 | 0.88 (0.82-0.95) | 0.0010 |
| Advanced fibrosis | 0.77 (0.57-1.02) | 0.0680 | 0.83 (0.71-0.97) | 0.0180 | 0.82 (0.62-1.09) | 0.1780 | 0.86 (0.73-1.01) | 0.0610 |

Abbreviations: OR, Odds ratio; CI, Confidence interval; MAFLD, metabolic (dysfunction)-associated fatty liver diseases.

Calorie Levels defined as (1=Q1, 2=Q2, 3=Q3, 4=Q4)

Carbohydrates Levels defined as (1=Q1, 2=Q2, 3=Q3, 4=Q4)

**Table S7** **Ordinal Logistic Regression for Improved Protein and Fat Intake Levels (in Quartiles), Stratified by the presence of MAFLD**

| Covariate | Protein | | | | Fat | | | |
| --- | --- | --- | --- | --- | --- | --- | --- | --- |
|  | MAFLD | | Non-MAFLD | | MAFLD | | Non-MAFLD | |
|  | OR (95% CI) | P-value | OR (95% CI) | P-value | OR (95% CI) | P-value | OR (95% CI) | P-value |
| Sarcopenia | 0.93 (0.74-1.18) | 0.5590 | 0.94 (0.76-1.18) | 0.6180 | 0.9 (0.71-1.14) | 0.3830 | 0.89 (0.71-1.11) | 0.2920 |
| Age | 0.97 (0.96-0.98) | <0.001 | 0.98 (0.98-0.98) | <0.001 | 0.97 (0.96-0.97) | <0.001 | 0.97 (0.97-0.98) | <0.001 |
| Male | 0.86 (0.74-1) | 0.0530 | 1.01 (0.94-1.09) | 0.7090 | 0.94 (0.81-1.1) | 0.4640 | 1.02 (0.94-1.1) | 0.6610 |
| Race |  |  |  |  |  |  |  |  |
| Black | Reference |  | Reference |  | Reference |  | Reference |  |
| Hispanic | 1.19 (0.97-1.46) | 0.0930 | 1.22 (1.1-1.34) | <0.001 | 0.8 (0.66-0.99) | 0.0370 | 0.86 (0.78-0.95) | 0.0030 |
| White | 1.4 (1.14-1.72) | 0.0010 | 1.2 (1.09-1.31) | <0.001 | 1.37 (1.11-1.69) | <0.001 | 1.32 (1.2-1.44) | <0.001 |
| Others | 1.54 (1.02-2.33) | 0.0390 | 1.27 (1.05-1.53) | 0.0150 | 0.66 (0.43-1) | 0.0490 | 0.58 (0.48-0.7) | <0.001 |
| Obesity |  |  |  |  |  |  |  |  |
| Normal | Reference |  | Reference |  | Reference |  | Reference |  |
| Overweight | 0.8 (0.6-1.06) | 0.1160 | 0.95 (0.87-1.03) | 0.2180 | 0.94 (0.71-1.25) | 0.6780 | 0.96 (0.88-1.04) | 0.2870 |
| Obese | 1.06 (0.8-1.41) | 0.6740 | 0.97 (0.87-1.07) | 0.5390 | 1.08 (0.82-1.44) | 0.5710 | 1.15 (1.04-1.28) | 0.0080 |
| Diabetes | 1.17 (0.95-1.44) | 0.1400 | 1.05 (0.88-1.24) | 0.5950 | 0.88 (0.72-1.09) | 0.2420 | 0.75 (0.63-0.89) | 0.0010 |
| Hypertension | 0.95 (0.81-1.11) | 0.5190 | 0.97 (0.88-1.06) | 0.4970 | 0.94 (0.8-1.11) | 0.4790 | 0.9 (0.81-0.99) | 0.0240 |
| Hypercholesterolemia | 1.32 (1.1-1.58) | 0.0030 | 1.09 (0.98-1.21) | 0.0980 | 1.03 (0.86-1.23) | 0.7580 | 0.93 (0.84-1.04) | 0.2040 |
| Smoking | 1.2 (1.03-1.4) | 0.0180 | 1.07 (0.99-1.16) | 0.0690 | 1.18 (1.01-1.37) | 0.0340 | 1.12 (1.04-1.21) | 0.0030 |
| Advanced fibrosis | 0.71 (0.54-0.94) | 0.0170 | 0.86 (0.74-1) | 0.0530 | 0.78 (0.59-1.03) | 0.0820 | 0.86 (0.74-1.01) | 0.0630 |

Abbreviations: OR, Odds ratio; CI, Confidence interval; MAFLD, metabolic (dysfunction)-associated fatty liver diseases.

Protein Levels defined as (1=Q1, 2=Q2, 3=Q3, 4=Q4)

Fat Levels defined as (1=Q1, 2=Q2, 3=Q3, 4=Q4)

**Table S8** **Hazard Ratios of Risk Factors of MAFLD Patients for All-cause Mortality, Stratified by Age**

| Covariate | Overall | | < 60 years | | ≥ 60 years | |
| --- | --- | --- | --- | --- | --- | --- |
|  | HR (95% CI) | P-value | HR (95% CI) | P-value | HR (95% CI) | P-value |
| Age | 1.08 (1.07-1.08) | < 0.001 | 1.07 (1.05-1.08) | < 0.001 | 1.09 (1.07-1.11) | < 0.001 |
| Male | 1.19 (1.04-1.35) | 0.01 | 1.15 (0.94-1.40) | 0.18 | 1.23 (1.04-1.48) | 0.016 |
| Race |  |  |  |  |  |  |
| Black | Reference |  | Reference |  | Reference |  |
| Hispanic | 0.65 (0.55-0.88) | < 0.001 | 0.63 (0.49-0.80) | < 0.001 | 0.66 (0.52-0.84) | < 0.001 |
| White | 0.88 (0.75-1.03) | 0.108 | 0.81 (0.64-1.03) | 0.088 | 0.93 (0.75-1.15) | 0.5 |
| Others | 0.46 (0.30-0.68) | < 0.001 | 0.37 (0.21-0.68) | 0.001 | 0.58 (0.33-1.01) | 0.-55 |
| Physical activity |  |  |  |  |  |  |
| Inactive | Reference |  | Reference |  | Reference |  |
| Insufficiently active | 0.85 (0.73-0.99) | 0.036 | 0.79 (0.63-0.99) | 0.038 | 0.90 (0.73-1.10) | 0.295 |
| Active | 0.79 (0.67-0.93) | 0.004 | 0.78 (0.60-1.00) | 0.052 | 0.78 (0.63-0.96) | 0.021 |
| Protein |  |  |  |  |  |  |
| < 1.2 g/kg | Reference |  | Reference |  | Reference |  |
| 1.2-1.5 g/kg | 1.25 (1.02-1.52) | 0.028 | 1.23 (0.94-1.63) | 0.137 | 1.28 (0.96-1.70) | 0.098 |
| ≥ 1.5g/kg | 0.79 (0.64-0.96) | 0.021 | 1.00 (0.75-1.33) | 0.978 | 0.64 (0.48-0.85) | 0.002 |
| Cirrhosis | 1.27 (1.05-1.52) | 0.012 | 1.33 (0.97-1.87) | 0.106 | 1.23 (0.99-1.54) | 0.059 |
| Diabetes | 1.84 (1.59-2.12) | < 0.001 | 1.94 (1.54-2.45) | < 0.001 | 1.83 (1.52-2.21) | < 0.001 |
| Hypertension | 1.27 (1.13-1.44) | < 0.001 | 1.41 (1.17-1.70) | < 0.001 | 1.18 (1.00-1.40) | 0.045 |
| Hypercholesterolemia | 0.88 (0.77-1.01) | 0.074 | 0.80 (0.63-1.00) | 0.051 | 0.98 (0.82-1.17) | 0.822 |
| Smoking | 1.45 (1.28-1.65) | < 0.001 | 1.44 (1.18-1.74) | < 0.001 | 1.47 (1.24-1.74) | < 0.001 |

Abbreviations: HR, Hazard ratio; CI, Confidence interval; MAFLD, metabolic (dysfunction)-associated fatty liver diseases.
