# Supplementary material for: Diet was less significant than physical activity in the prognosis of people with sarcopenia and metabolic dysfunction-associated fatty liver diseases: Analysis of the National Health and Nutrition Examination Survey III
Source: Front Endocrinol (Lausanne). 2023 Feb 23;14:1101892. doi: 10.3389/fendo.2023.1101892 (PMC9995978; doi:10.3389/fendo.2023.1101892)
Supplement: Supplementary file 2 [file DataSheet_2.docx]

**Supplementary Material**

**Methods**

HBV was defined as hepatitis B surface antigen-positive. HCV was defined who tested positive for the hepatitis C antibody or HCV RNA by PCR.

Information on demographic characteristics (such as age, sex, race/ethnicity, educational levels), lifestyle (i.e. smoking status, physical activity, excess alcohol consumption, diet), and prior medical conditions (CVD and any cancer) was collected at baseline through self-reports.

Serum creatinine was measured using a kinetic rate Jaffe method in NHANES III, recalibrated to standardized creatinine measurements obtained at the Cleveland Clinic Research Laboratory (Cleveland, OH) as standard creatinine = – 0.184 + 0.960 × NHANES III–measured serum creatinine (1). eGFR was estimated as 175 × (standardized serum creatinine) -1.154 × (age)-0.203 × 0.742 (if the individual is woman) × 1.212 (if the individual is African American) (2). Patients with eGFR < 60 ml/min per 1.73 m2 were identified as chronic kidney diseases.

The activities are classified into moderate (METs ranging from 3 to 6) and vigorous (METs above 6) categories based on their intensity rates. Active group was characterized as those who engaged in moderate or vigorous activity at least five or three times per week. The inactive group was defined as those who participate in no physical activity during their leisure time. The insufficiently active group fell in the middle between active and inactive levels of physical activity(3).

NHANES III questionnaire on exercise includes: walking activity, jog or run activity, bicycle activity, swim activity, aerobics activity, other dancing activity, calisthenics activity, garden/yard activity, lift weights activity, and other activities with self-reported intensity.

Moderate activity (METs ranging from 3 to 6) includes: walking activity, bicycle activity, swim activity, aerobics activity, other dancing activity, calisthenics activity, garden/yard activity, lift weights activity.

Vigorous activity (METs above 6) includes: jog or run activity.

Other self-reported activities were also included and classified into above groups according to their MET levels.

**Figure Legends**

Figure 1. Flow Chart of Participants for the Study

Figure 2. Prevalence of Sarcopenia among Participants with and without MAFLD

**Tables**

Table 1. Multivariate Analysis for Sarcopenia in overall population

Table 2. Age, Sex and Race-adjusted Odds Ratio (OR) for Sarcopenia in patients with and without MAFLD

Table 3. Hazard Ratios of Risk Factors for All-cause Mortality (Multiple Imputation Analysis)

Table 4. Hazard Ratios of Risk Factors of Patients with Sarcopenia for All-cause Mortality, Stratified by Age

Supplementary Table 1. Demographic, Dietary, and Physical Activity Characteristics of Participants Categorized by the Presence of MAFLD and Sarcopenia

Supplementary Table 2. Laboratory Characteristics of Participants Categorized by the Presence of MAFLD and Sarcopenia

Supplementary Table 3. Prevalence of Sarcopenia in MAFLD and Non-MAFLD Participants

Supplementary Table 4. Ordinal Logistic Regression for Improved Physical Activity Levels, Stratified by the presence of MAFLD

Supplementary Table 5. Ordinal Logistic Regression for Improved Protein Intake Levels, Stratified by the presence of MAFLD

Supplementary Table 6. Ordinal Logistic Regression for Improved Calorie and Carbohydrates Intake Levels (in Quartiles), Stratified by the presence of MAFLD

Supplementary Table 7. Ordinal Logistic Regression for Improved Protein and Fat Intake Levels (in Quartiles), Stratified by the presence of MAFLD

Supplementary Table 8. Hazard Ratios of Risk Factors of MAFLD Patients for All-cause Mortality, Stratified by Age

1. Coresh J, Selvin E, Stevens LA, Manzi J, Kusek JW, Eggers P, et al. Prevalence of Chronic Kidney Disease in the United States. *Jama* (2007) 298(17):2038-47. Epub 2007/11/08. doi: 10.1001/jama.298.17.2038.

2. Levey AS, Coresh J, Greene T, Stevens LA, Zhang YL, Hendriksen S, et al. Using Standardized Serum Creatinine Values in the Modification of Diet in Renal Disease Study Equation for Estimating Glomerular Filtration Rate. *Ann Intern Med* (2006) 145(4):247-54. Epub 2006/08/16. doi: 10.7326/0003-4819-145-4-200608150-00004.

3. Pate RR, Pratt M, Blair SN, Haskell WL, Macera CA, Bouchard C, et al. Physical Activity and Public Health. A Recommendation from the Centers for Disease Control and Prevention and the American College of Sports Medicine. *Jama* (1995) 273(5):402-7. Epub 1995/02/01. doi: 10.1001/jama.273.5.402.
